# Single-step precision programming of decoupled multiresponsive soft millirobots

**DOI:** 10.1073/pnas.2320386121

**Published:** 2024-03-21

**Authors:** Zhiqiang Zheng, Jie Han, Qing Shi, Sinan Ozgun Demir, Weitao Jiang, Metin Sitti

**Affiliations:** ^a^Physical Intelligence Department, Max Planck Institute for Intelligent Systems, Stuttgart 70569, Germany; ^b^State Key Laboratory for Manufacturing Systems Engineering, Xi’an Jiaotong University, Xi’an 710054, China; ^c^School of Mechanical Engineering, Xi’an Jiaotong University, Xi’an 710054, China; ^d^Intelligent Robotics Institute, School of Mechatronical Engineering, Beijing Institute of Technology, Beijing 100081, China; ^e^Key Laboratory of Biomimetic Robots and Systems (Beijing Institute of Technology), Ministry of Education, Beijing 100081, China; ^f^Institute for Biomedical Engineering, ETH Zurich, Zurich 8092, Switzerland; ^g^School of Medicine and College of Engineering, Koç University, Istanbul 34450, Turkey

**Keywords:** soft robotics, soft electronics, environmental response, miniature robot

## Abstract

Stimuli-responsive soft robots offer new capabilities for the fields of medical and rehabilitation robotics, artificial intelligence, and soft electronics. Precisely programming the shape morphing and decoupling the multiresponsiveness of such robots is crucial to enable them with ample degrees of freedom and multifunctionality, while ensuring high fabrication accuracy. However, current designs featuring coupled multiresponsiveness or intricate assembly processes face limitations in executing complex transformations and suffer from a lack of precision. Therefore, we propose a one-stepped strategy to program multistep shape-morphing soft millirobots (MSSMs) in response to decoupled environmental stimuli. Our approach involves employing a multilayered elastomer and laser scanning technology to selectively process the structure of MSSMs, achieving a minimum machining precision of 30 μm. The resulting MSSMs are capable of imitating the shape morphing of plants and hand gestures and resemble kirigami, pop-up, and bistable structures. The decoupled multistimuli responsiveness of the MSSMs allows them to conduct shape morphing during locomotion, perform logic circuit control, and remotely repair circuits in response to humidity, temperature, and magnetic field. This strategy presents a paradigm for the effective design and fabrication of untethered soft miniature robots with physical intelligence, advancing the decoupled multiresponsive materials through modular tailoring of robotic body structures and properties to suit specific applications.

## Significance

This paper introduces a single-step methodology for crafting soft millirobots capable of intricate multistep shape morphing in response to independently decoupled environmental stimuli. The unique feature of decoupling shape morphing empowers the independent programming of each transformation step, resulting in a substantial augmentation of both degrees of freedom and overall system functionality. Our approach facilitates the realization of multistep shape morphing, giving rise to a myriad of intricate three-dimensional (3D) structures, encompassing biomimetic shapes, expressive gestures, kirigami architectures, pop-ups, and bistable configurations. This technique embodies a versatile paradigm for crafting multifunctional and adaptable 3D devices, applicable across a diverse spectrum of fields.

Design and fabrication of soft robots that imitate the capabilities of living organisms have been a key in robot design, fabrication, and control (1, 2). Living creatures possess feedback mechanisms that grant them sensitive perception, sophisticated and agile movement, and adaptability to their surroundings (3, 4). Soft robots, utilizing materials with similar properties, have demonstrated significant physical adaptability in various applications (5), such as human-machine interfaces (6, 7), bioinspired robotics (8, 9), medical devices (10, 11), and exploration (12). However, compared to rigid robots, current soft robots often exhibit limited dexterity, precision, complex assembly process, and autonomous performance (13, 14). To overcome these limitations, it is vital to develop efficient fabrication methods that can seamlessly integrate active materials and heterogeneous structures that respond to various stimuli (15–17), leading to enhanced performance and functionality in soft robots (18, 19).

Responsive shape morphing plays a crucial role in achieving advanced performance and functions of soft robots (20, 21), as it enables them to adapt to various environmental stimuli and perform complex tasks (22, 23). Unlike traditional rigid robots, soft robots are made of flexible materials and can move in ways that are challenging to control precisely (13, 21, 24). Shape morphing offers a promising strategy for transforming two-dimensional (2D) electronics into three-dimensional (3D) structured devices (25, 26). This technique employs responsive materials that undergo heterogeneous expansion or compression, generating internal stress to induce shape changes (27). Successful applications of this technique include the creation of 3D electrodes using responsive bilayer polymers (28–32), patterned polymers (30, 33), liquid crystal polymers (34–36), and polymer films with gradient structures (37–39) or assembled architectures (40). However, current fabrication methods always involve arduous assembly processes (40–44), design inflexibility constraint (16, 45–48), and insufficient precision (18, 49, 50). Hence, a necessity arises for the development of a universally applicable fabrication methodology characterized by programmability, high precision, and simplicity. Such a method would serve as a versatile approach for producing multifunctional and flexible 3D devices, applicable across a spectrum of diverse applications.

**Fig. 1.**
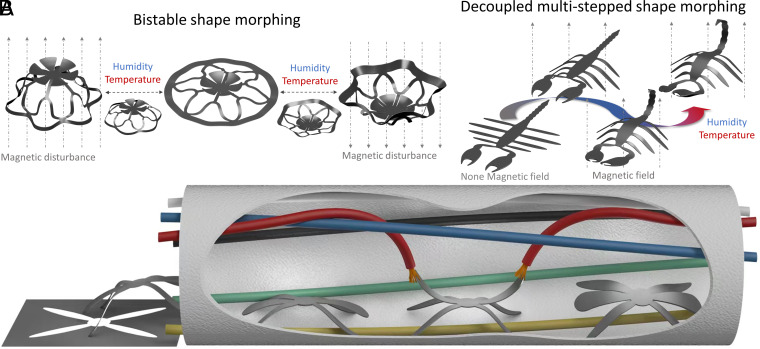
Principle of bistable and decoupled shape morphing and illustration of the circuit required for multistep shape-morphing soft millirobots (MSSMs). (*A*) In the bistable structure, the magnetic disturbance is utilized to manipulate the deformation pattern, while humidity and temperature can reset the structure to a flat state. In the scorpion-shaped structure, the legs, arms and tail modules of the robotic body can respond separately to magnetic fields and humidity, ensuring uninterrupted and targeted responses. (*B*) The schematic illustrates the MSSM within a circuit pipeline. The MSSM exhibits high mobility, enabling it to navigate through narrow environments, while its robotic arms are capable of repairing circuits.

Here, we introduce a one-step technique for programming and decoupling multistep shape morphing in response to different environmental stimuli, allowing for the complex 3D shape morphing. The unique feature of decoupling shape morphing allows for independent programming of each transformation step in response to specific stimuli, augmenting the degrees of freedom (DOF) and overall functionality of the system. Utilizing xerogel, laser-induced graphene (LIG), and whole-material laser patterning, our approach enables multistep shape morphing, creating diverse structures with intricate 3D morphologies, including biomimic shapes, gestures, kirigami architectures, pop-ups, and bistable structures. To elucidate the underlying mechanism, we developed a theoretical model and employed finite element analysis (FEA) to predict and design the shape-morphing behavior. As a proof-of-concept, we demonstrate the continuous transformation of bistable structures, showcasing a scorpion-shaped locomotion (Fig. 1*A*). Leveraging their high programmability, the MSSMs (multistep shape-morphing soft millirobots) serve as environmentally adaptive switches for logic control circuits and function as maintenance robots for circuit repair, capitalizing on their decoupled shape morphing capabilities (Fig. 1*B*). The exceptional programmability and decoupled nature of these MSSMs pave the way for the development of multifunctional and physically intelligent robots, with potential applications across diverse fields.

## Results

### Decoupling and Reprocessing of Programmable Shape Morphing by Xerogel Patterning.

The fabrication process of an MSSM was shown in *SI Appendix*, Fig. S1. The multilayer structure was composed of alginate xerogel, LIG, and magnetic elastomer, and prestress was introduced between the xerogel and elastomer layers during the transformation of hydrogel into xerogel. After patterning the desired shape, the prestress could be released through multiple steam humidification-drying cycles, leading to a stress-induced 2D to 3D structural transformation, termed the “activation of MSSM.” The rapid and reversible humidity-induced shape-morphing of MSSM was attributed to the swelling process of the xerogel layer, which exhibited high sensitivity to relative humidity (RH).

**Fig. 2.**
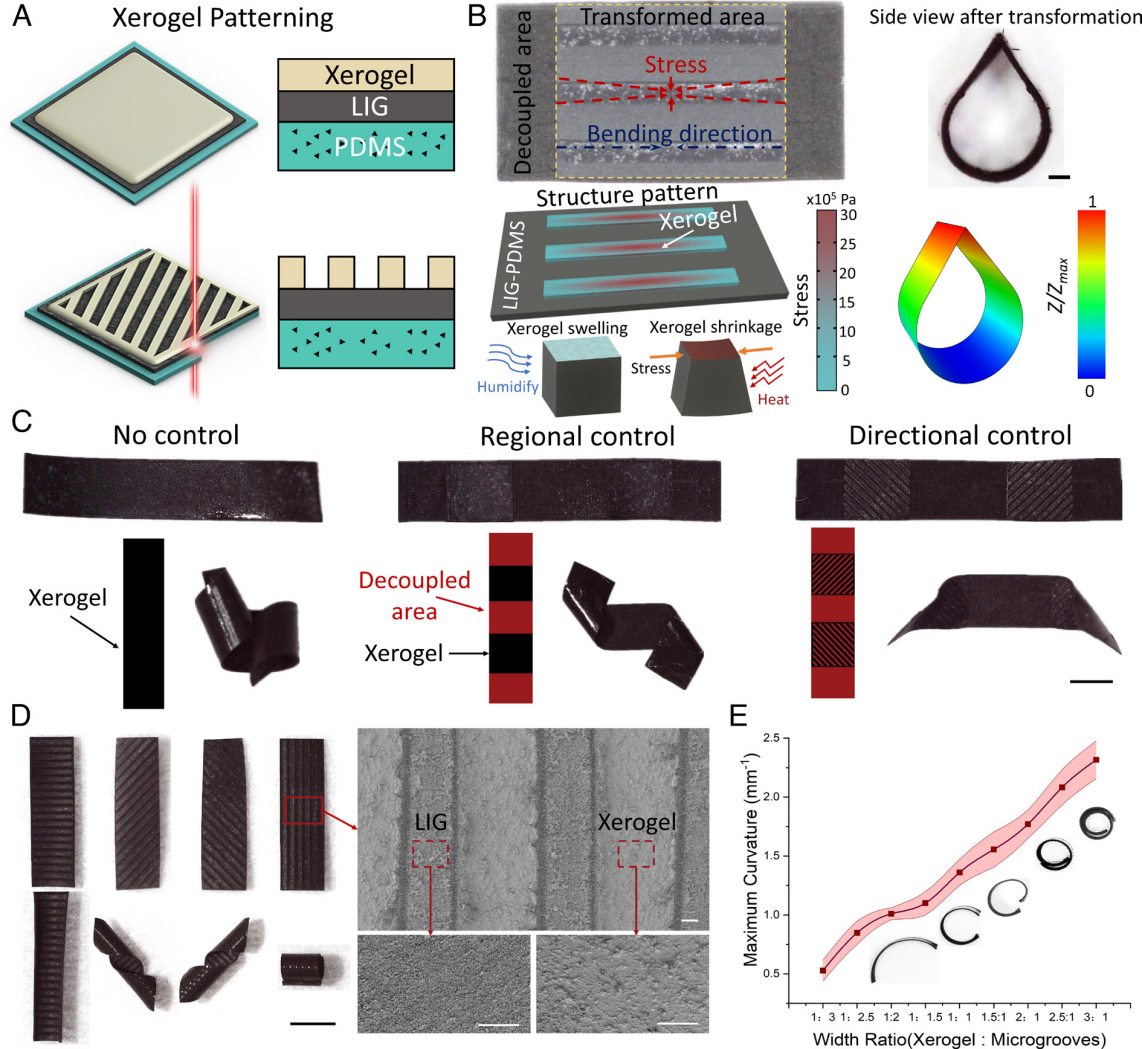
Decoupling, programming and reprocessing of MSSM shape morphing. (*A*) Schematic illustration of xerogel patterning by laser raster. (*B*) The structure design, FEA, and mechanism of beam shape morphing with partially removing xerogel in response to the humidity changes. (*C*) The reprocessing process based on laser patterning on the xerogel layer. (Scale bar: 1 mm.) (*D*) Various bending direction of beam structure based on the xerogel laser patterning. Scale bar: 3 mm (black) and 50 μm (white). (*E*) Width ratio (xerogel area: nonxerogel area) of beam structures with different maximum bending curvature. Each dot represents the average curvature for three different stripe structures under a width ratio (xerogel area: microgrooves area) change with stripe shapes ± standard error of the mean (SEM) (n = 3).

Upon exposure to humid environments, the xerogel layer undergoes rapid water absorption, resulting in a substantial increase in volume. This leads to the opening of the MSSM from its initially closed state under ambient conditions. This reversible shape-morphing process can be achieved by manipulating humidity, temperature, and chemicals in the environment. Since temperature is one of the key base quantities, this method can only decouple magnetic field and the combination of humidity and temperature. To achieve diverse 3D structures suitable for various applications, we can decouple, program, and reprocess the stimuli-responsive shape morphing of MSSMs by patterning the xerogel layer, as illustrated in Fig. 2*A*. This design approach provides the flexibility to tailor the shape-morphing behavior of MSSMs for specific functions. Fig. 2*B* demonstrates the selective and precise removal of the xerogel layer through laser patterning in the raster mode, which enables the adjustment of stress distributions and angles that induced by the humidity-responsive layer. In a high-humidity environment, the beam structure would be in the flat state and the xerogel would swell by absorbing water. In a low-humidity environment, the beam structure assumes a bending state as the xerogel undergoes shrinkage through water evaporation. Throughout the processes of xerogel shrinkage and swelling, the change in length of the xerogel rectangle exceeds that of its width, given the uniform hygroscopic expansion across the entire xerogel area. As a result, the bending direction consistently aligns along the longitudinal axis of the xerogel rectangle.

In the simulation results, *Z* and *Z*_max_ represented the displacement and maximum displacement along the *Z*-axis, respectively. Moreover, this approach also allowed for the reprocessing of the shape morphing, as shown in Fig. 2*C*. The nonpatterned beam structure (3 mm × 15 mm, length by width) exhibited an asymmetric shape morphing with a fully covered xerogel layer, which was due to the inhomogeneous stress distribution introduced during the electro-crosslinked hydrogel and hydrogel-xerogel transition process. By removing the xerogel layer in the middle and two ends, the beam deformation was programmed into an S shape with a controlled active and inactive region. Furthermore, based on the previous design, a grid pattern of xerogel with a specific angle of 45° was occupied to tune the bending direction into the desired structure, as depicted in Fig. 2*D* and Movie S1. The detailed mechanism and mechanical analysis were shown in the supplementary material titled “mechanical analysis.”

Based on mechanical analysis, we could establish the relationship between the curvature and width ratio. Scanning electron microscopic images provided a detailed surface outlook before and after laser patterning. The fast and precise patterning approach enabled the decoupling and programming of the stimuli-responsive shape morphing of MSSMs. As the xerogel layer functioned as the active layer to provide stress in humidity-responsive shape morphing, the area ratio of the xerogel region played a crucial role in determining the MSSM’s shape-morphing ability. In Fig. 2*E*, we patterned the beam structure with different width ratios of xerogel and microgroove (xerogel-removed area). It was shown that the maximum bending curvature was 2.3 mm^−1^ when the width ratio was 3:1 (xerogel cover ratio, 75%), and it decreased to 0.5 mm^−1^ when the width ratio changed to 1:3 (xerogel cover ratio, 25%).

### Programming Shape Morphing Based on LIG Layer Patterning.

**Fig. 3.**
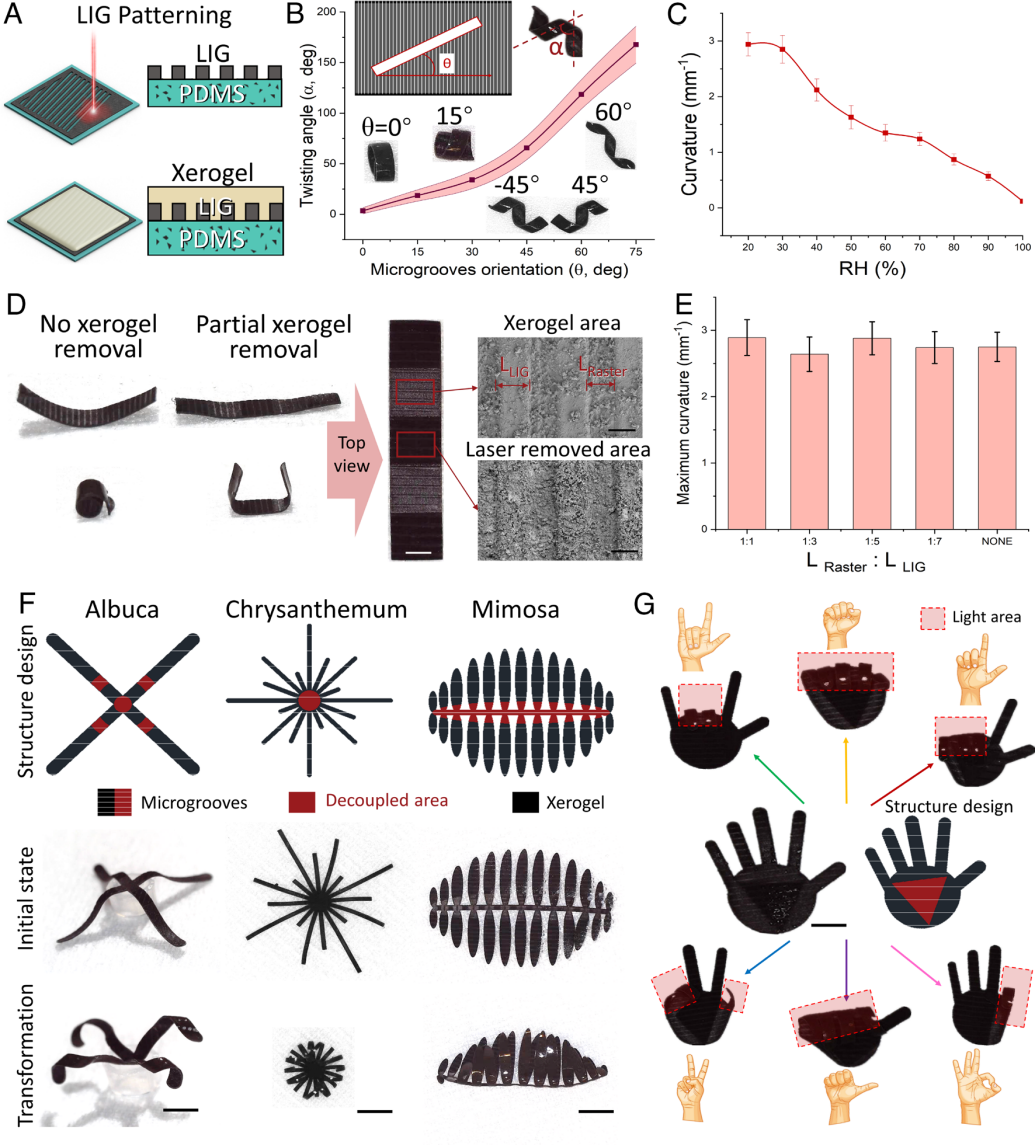
Laser-induced graphene (LIG) layer patterning guiding the MSSM shape morphing programming. (*A*) Schematic illustration of LIG patterning by laser raster. (*B*) Twisting angle (α) of strip structures with different microgroove orientation (θ). Each dot represents the average twisting angle for three different stripe structures under a microgroove orientation changing with stripe shapes ± SEM (n = 3). (*C*) Curvature of strip structures with different RH value. Each dot represents the average curvature for three different stripe structures under a RH change with stripe shapes ± SEM (n = 3). (*D*) The shape morphing of the structure with partially removing xerogel in response to the humidity changes and the detailed surface structure. Scale bar: 1 mm (white) and 100 μm (black). (*E*) Maximum curvature of strip structures with different ration of L_Raster_ and L_LIG_. Each column represents the average curvature for three different stripe structures under a L_Raster_: L_LIG_ change with stripe shapes ± SEM (n = 3). (*F*) The shape morphing of the biomimetic structures including chrysanthemum, albuca, and mimosa. (Scale bar: 1 mm.) (*G*) The shape morphing of the hand structures in response to the regional light heating. (Scale bar: 1 mm.)

Except for the xerogel patterning, we could also pattern the LIG layer to program the humidity-induced shape morphing. The fabrication method and detailed process are shown in Fig. 3*A* and *SI Appendix*, Fig. S2. Before electrodeposition, a raster mode UV-laser was employed to generate a grid-patterned LIG on the surface of the magnetic elastomer. The grid-patterned LIG served as a directional transformation electrode, guiding the electrodeposition process. Additionally, by increasing the laser power, the substrate stiffness could be simultaneously adjusted. Based on the LIG layer patterning, there were stiffness and xerogel gradients along a certain direction, which could be used to guide the bending direction. In *SI Appendix*, Fig. S3, the stiffness of strip structure with dimensions of 10 mm × 4 mm (length by width) was tested in the environment of RH 30% and temperature 20 °C. After the calculation, the stiff of the beam structure is 25.76 mN/mm^2^. *SI Appendix*, Fig. S4 showed the maximum bending force of this strip structure reached 51.3 mN in the environment of RH 28% and temperature 30 °C. The xerogel removal also helped to achieve more complex structural shape morphing.

In Fig. 3*B*, a series of strip structures with dimensions of 16 mm × 2 mm (length by width, aspect ratio of eight) were laser cut along different microgroove orientations (*θ*) relative to the LIG grid pattern. This process resulted in the generation of various twisting angles (*α*) in the deformed structure. The experiment images and Movie S2 showcased the deformation results of the strip structure with different microgroove orientations, namely 0°, 15°, 45°, −45°, and 60°. In the LIG pattern method, where no xerogel removal occurred, no obvious loss in bending capability was observed. The maximum curvature, as depicted in Fig. 3*C*, reached approximately 3 mm^−1^. In Fig. 3*D*, the LIG-patterned structure perfectly bent into a spiral structure without any further treatment (*θ* = 0°).

Besides, laser engraving can also be utilized to reprogram the shape morphing by removing part of the xerogel area as illustrate in Movie S3. In the detailed surface structure (Fig. 3*D*), the microgrooves of LIG can be seen. The length ratio of LIG area (L_LIG_) and laser rasterized area (L_Raster_) had little effect on the maximum curvature. However, a smaller length ratio (L_Raster_: L_LIG_) was less efficient in guiding the bending direction (Fig. 3*E*). In Fig. 3*F* and Movie S4, we generated a series of 2D structures to conduct programmable shape morphing to mimic natural plants, such as chrysanthemum, albuca, and mimosa, in response to humidity and temperature. In these plants, LIG microgrooves can guide the bending direction without the need for programming the xerogel layer, thereby preserving the overall structural integrity. In Fig. 3*G* and Movie S5, we developed a hand structure that can present different gestures through regional light heating (power density, 20 mW/mm^2^). In this design, the xerogel area acted as muscle that can be selectively activated by light to conduct various bending motions.

### Complex Shape Morphing Programming Based on Kirigami Structure.

**Fig. 4.**
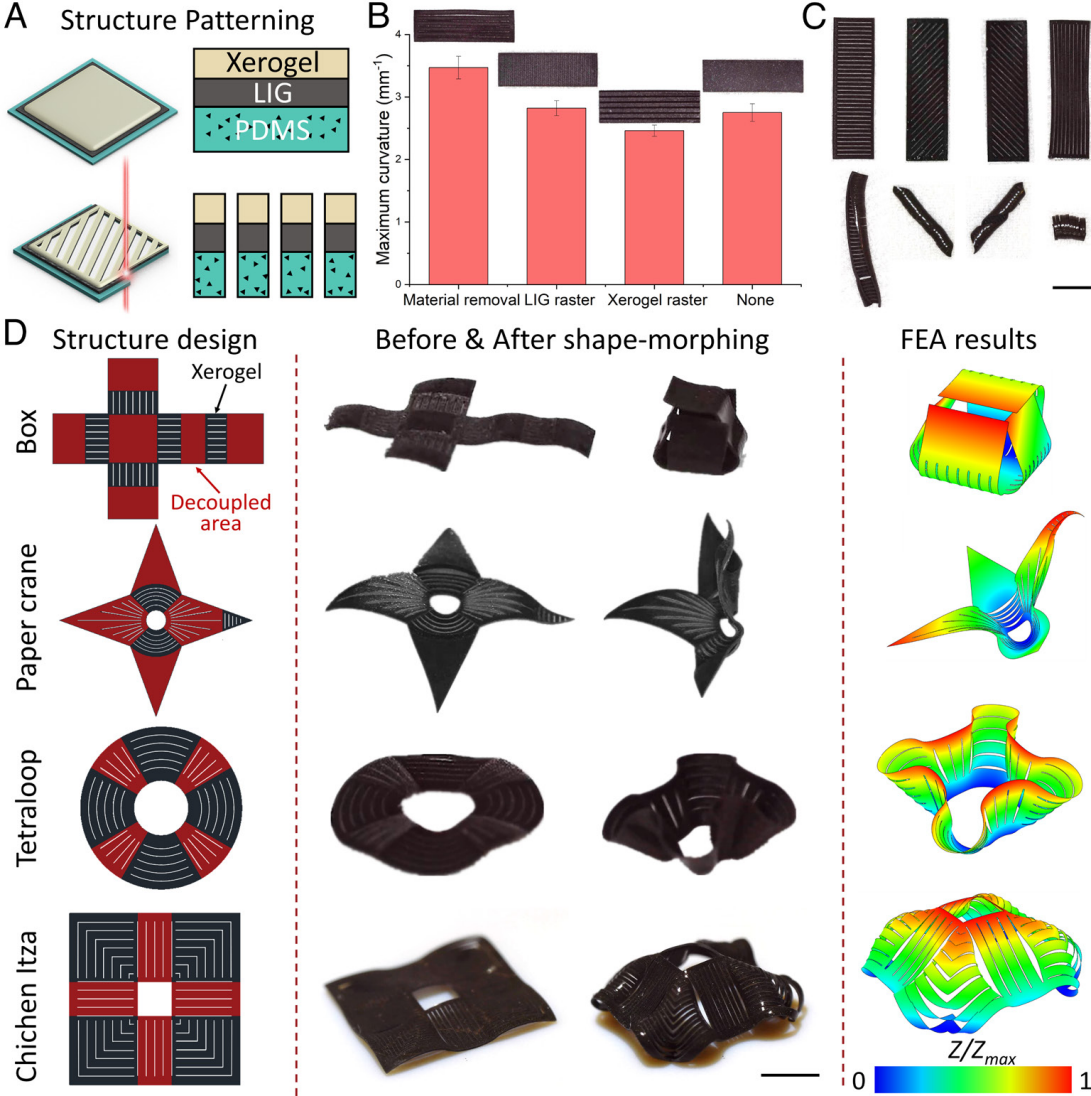
Complex shape morphing programming demonstrations. (*A*) Schematic illustration of laser-induced material removal. (*B*) Maximum curvature of strip structures with different fabrication processes including material removal, LIG layer raster, and xerogel layer raster without treatment. Each column represents the average curvature for five different stripe structures fabricated by different fabrication methods with stripe shapes ± SEM, n = 5. (*C*) Various bending direction of beam structure based on the whole material laser patterning. (Scale bar: 3 mm.) (*D*) The demonstrations and simulation results of various complex shape morphing kirigami structures including box, paper crane, tetraloop, and Chichen Itza. (Scale bar: 1 mm.)

To enhance programming flexibility with greater bending ability, we utilized laser patterning to create kirigami structures by cutting through the entire material, as depicted in Fig. 4*A*. Fig. 4*B* demonstrated that the structure with the entire material removed exhibited a larger bending curvature compared to structures with LIG rasterization, xerogel removal, or no treatment. The transformation speed and stability tests are shown in *SI Appendix*, Fig. S5. The strip structures can complete the transformation in 10 s in RH 30% and temperature higher than 50 °C. Besides, the strip structure can maintain the transformation ability after eight times cycled shape morphing. And after exposure in RH 90% for 12 h, the strip structure retains its transformation ability. The diminished stiffness in a particular direction resulted from the comprehensive removal of material. Consequently, we could program shape morphing based on this fabrication strategy, as shown in Fig. 4*C* and Movie S6. To predict shape morphing, we conducted FEA utilizing our xerogel and structure patterns, as presented in *SI Appendix*, Fig. S6. The box, paper crane, tetraloop, and Chichen Itza structures, shown in Fig. 4*D* and Movie S7, perfectly matched our simulation results. In the design of the kirigami structures, the black area represented xerogel, while the white areas represented voids. The red areas indicated the LIG layer without the xerogel layer. Additional complex structures, including the tetrahedron, butterfly, and tricyclic shapes, were depicted in *SI Appendix*, Fig. S7. In the simulation results, *Z* and Z_max_ represented the displacement and maximum displacement along the *Z*-axis, respectively.

### Bistable and Pop-Up Structure Actuated by Decoupled Multiple Stimuli.

**Fig. 5.**
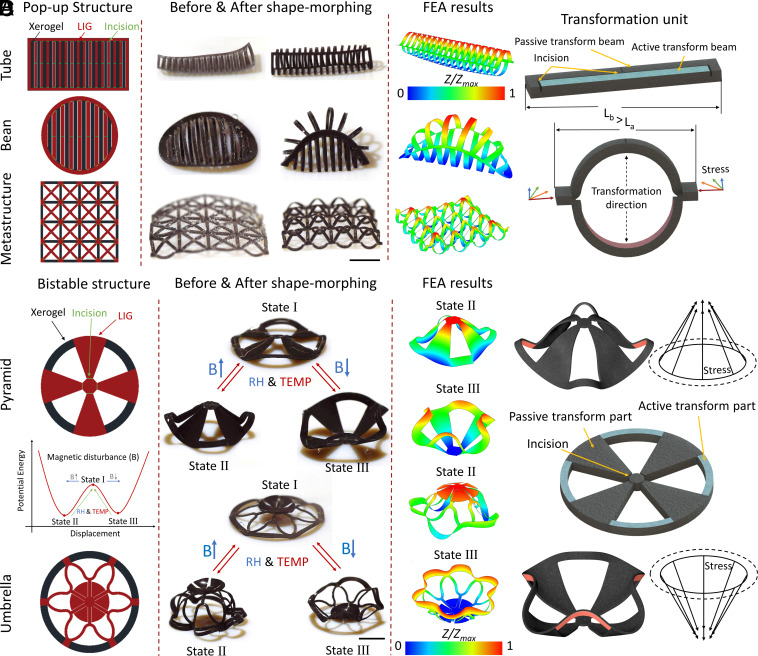
Bistable structures actuated by decoupled multiple stimuli. (*A*) The design, deformation results, and the finite element analysis (FEA) results of a tube structure, bean structure, and metastructure. (Scale bar: 2 mm.) (*B*) The schematic illustrates the shape and stress change the pop-up structure unit during the transformation. (*C*) The design, mechanism diagram, deformation results, and the FEA results of pyramid- and umbrella-like bistable structures. (Scale bar: 2 mm.) (*D*) The schematic illustrates the stress and shape-changing process in the different morphing directions.

In addition to complete material removal, we also explored the generation of soft joints in specific areas by incorporating incisions. The detailed structure of these incisions was illustrated in *SI Appendix*, Fig. S8. Utilizing these incisions, we designed various pop-up structures, including tubes, beans, and metastructures. In Fig. 5*A* and Movie S8, the black, red, and green lines represented the xerogel area, the xerogel-removed area, and the incisions separately. In such pop-up structure design, the xerogel part had no incision and bent downward. On the other hand, the LIG part had no xerogel and underwent passive transformation through the joints with incisions, which bent in the opposite direction of the xerogel part. Fig. 5*B* shows that the pop-up structures transfer the linear (horizontal) shrinking stress into 2D (both vertical and horizontal) stress. The incisions acted as mono-directional joints to guide the bending directions (upward or downward).

Based on these pop-up structures, we could incorporate magnetic control to enrich the DOF to generate bistable structures in response to the decoupled multistimuli, as illustrated in Fig. 5*C* and Movie S9. The magnetic profile of the bistable structures was shown in *SI Appendix*, Fig. S9. These bistable structures exhibited two bending directions, which were controlled by the direction of the magnetic field. In a high RH environment, the structures were in state I, with relatively high potential energy, providing equal opportunities for transformation into states II and III. By decreasing the RH and applying a small magnetic impulse (10 mT uniform field) while in state I, the transformation direction could be changed. As state I was unstable under low RH conditions, a slight external force could easily guide the structure toward the state with the lowest potential energy. Fig. 5*D* showed that the bistable structures transfer the linear (circumferential) shrinking stress into 3D conical stress. This linear shrinking stress shortens the circumference, pushing up the passive parts through the joints. These bistable structures allow the incisions to bend in both upward and downward directions, which can be tuned by the external magnetic field. Consequently, we had developed a switch that automatically reset the structure in response to the RH and temperature of the environment, with the switch direction tunable through the application of a magnetic field.

### Locomotion and Shape Morphing of Scorpion-Shaped MSSM.

**Fig. 6.**
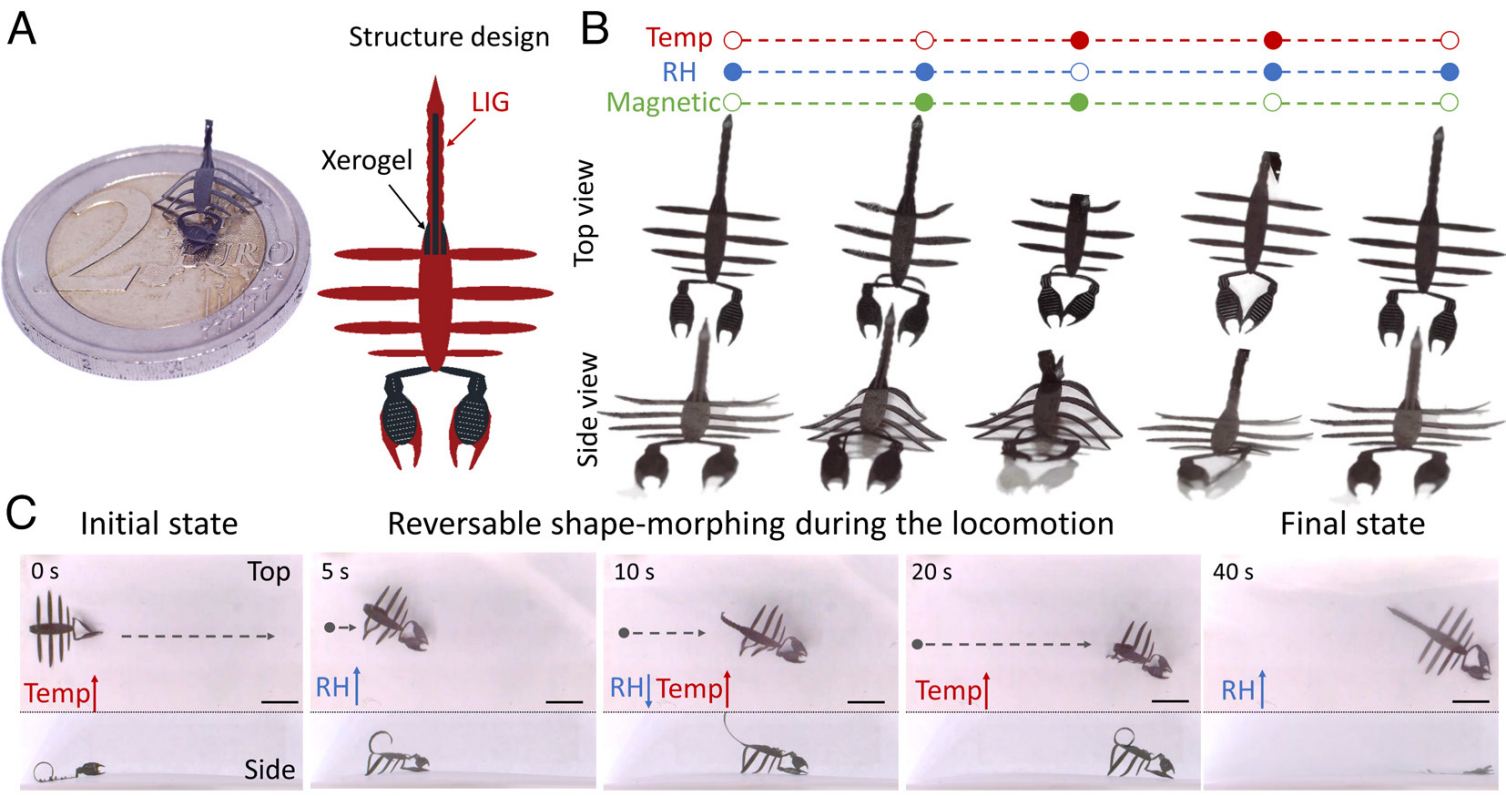
Scorpion-shaped MSSM actuated by decoupled multiple stimuli. (*A*) The size scale and structure design of the scorpion MSSM. (*B*) The shape morphing results in response to decoupled temperature, RH, and magnetic field stimuli. (*C*) Optical images from *Top* (*Top* part in each row) and side (*Bottom* part in each row) view of the MSSM walking on a substrate in air and conduct the transformation during the locomotion. (Scale bar: 3 mm.)

Taking inspiration from nature, where animals often distribute functions to different parts of their bodies (e.g., using legs for running and hands for grasping), we illustrate in Fig. 6 how the scorpion serves as an example of separated functionalities within the robotic body, with no interference. Fig. 6*A* demonstrated the size and structure design of the scorpion MSSM, where the black and red represented the xerogel area and the xerogel-removed area, respectively. The minimum sub-structure width was 30 μm. The pincers and tail areas of the scorpion responded to changes in RH and temperature through the xerogel material, enabling shape morphing, as illustrated in Fig. 6*B*. In contrast, the eight legs, not covered by xerogel, were exclusively actuated by the applied magnetic field. This design allowed the scorpion MSSM to undergo shape transformation during locomotion. To move the scorpion-shaped robot within the environment as in Fig. 6*C*, the legs were actuated by modulating the external magnetic field, following the signal defined in *SI Appendix*, Fig. S10. In order to generate the desired motion, a uniform magnetic field was applied in the X–Z plane, with the X-axis aligned in the intended direction of movement. This tilted the body forward, ensuring that only the leg tips made contact with the surface. Simultaneously, a sinusoidal magnetic field was applied perpendicular to the uniform field, inducing a left-to-right oscillatory motion. This design allowed the scorpion MSSM to exhibit locomotion capabilities while maintaining separate functionalities between the legs, pincers, and tail. The xerogel-based shape morphing enabled environmental responsiveness, while the magnetic actuation controlled the leg movement for locomotion.

### Electronic Application Demonstration of Multifunctional MSSMs.

**Fig. 7.**
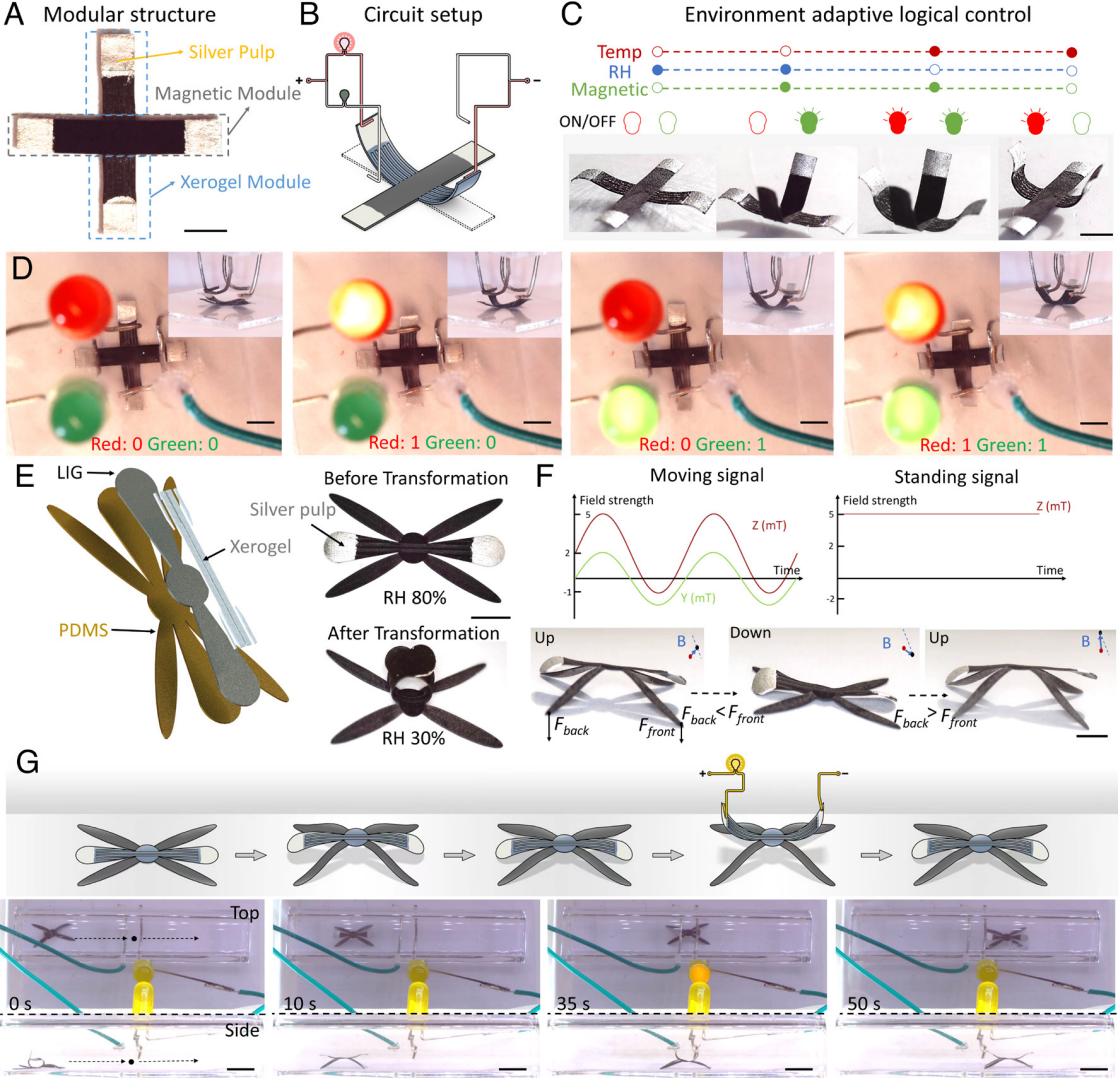
Soft electronic MSSMs with multistage shape morphing. (*A*) The modular design of the MSSM system allows it to function as an environmentally adaptive switch. (Scale bar: 2 mm.) (*B*) Illustration of the circuit setup used in the experiment. (*C*) Logic control of the environmental adaptive switch, responding to RH, temperature, and magnetic field inputs. (*D*) Control of red and green bulbs through decoupled shape morphing of the MSSM. (Scale bar: 2 mm.) (*E*) The MSSM’s structure, composition, and shape morphing in response to changes in RH. (Scale bar: 1 mm.) (*F*) The locomotion mechanism of the MSSM driven by magnetic field actuation signals. (Scale bar: 1 mm.) (*G*) Schematic representation of the MSSM repairing a circuit, and optical images of the MSSM navigating through a narrow pipe and connecting the circuit. *Top* and side views shown. (Scale bar: 3 mm.)

We demonstrated an untethered MSSM capable of electronic applications with in situ shape reconfigurability in various working environments, including RH, temperature, and magnetic fields, as shown in Fig. 7. This unique feature allowed the MSSM to function as a self-actuated circuit switch. In Fig. 7*A*, we divided the MSSM into two distinct modules: the magnetic module (responsive to magnetic fields) and the xerogel module (responsive to RH and temperature). To ensure efficient circuit connection, we used silver pulp to reduce contact resistance. Detailed structural design, magnetic profiles, and sheet resistance before electrodeposition and after xerogel removal can be found in *SI Appendix*, Figs. S11 and S12. Fig. 7*B* illustrated the circuit setup where the MSSM made contact with a wire and connected bulbs of different colors in response to different stimuli. There were four different configurations of the MSSM depicted in Fig. 7*C*, each producing different signals. In a high RH environment, the MSSM remained flat, and both bulbs remained off. In a high RH environment with a magnetic field, the magnetic module bent, turning on the green bulb. In a high-temperature environment with a magnetic field, both the xerogel and magnetic modules bent, resulting in the activation of both bulbs. When the magnetic field was removed, only the xerogel module bent, and the red bulb turned on. A detailed process explaining the adaptive logical control based on environmental conditions was presented in Fig. 7*D* and Movie S11. As a result of the point–point interaction between the MSSM and the environmental disturbance, the connection is unstable. Therefore, the connection area can be coated with liquid metal or another semi-liquid conductor to increase the contact area and reduce the shake.

Apart from its adaptive switch functionality, the MSSM could also serve as a circuit maintenance robot, as depicted in Fig. 7*E*. In this illustration, the LIG, xerogel, and PDMS layers were represented by black, gray, and orange colors, respectively. The MSSM’s arms retained their shape morphing ability in response to RH and temperature changes. Detailed structural designs and magnetic profiles for this configuration were shown in *SI Appendix*, Fig. S13. Fig. 7*F* presents the mechanism and magnetic control of the MSSM’s locomotion. The locomotion mechanism resembled that of a caterpillar and involved two steps: the up–down and down–up motion in a cyclical pattern. During the up–down phase, the robot stood up and tilted forward, transferring its body weight onto the front legs, resulting in a larger force on the front legs (F_front_) compared to the back legs (F_back_). After reaching a certain point, the friction acting on the back legs decreased, causing them to be pulled forward. In the down–up process, the body weight shifted backward, exerting a larger force on the back legs (F_back_ > F_front_) and propelling the front legs forward along the body’s long axis. This enabled the MSSM to navigate narrow pipes and autonomously repair damaged circuits, as demonstrated in Fig. 7*G* and Movie S12. Due to decoupled control, the MSSM exhibited two-step shape morphing, allowing it to grip the circuit in a stand-up configuration. The stand-up configuration was controlled by the magnetic field, while the grasping motion was influenced by changes in RH and temperature.

## Discussion

In this study, we have introduced a straightforward methodology aimed at decoupling multistep shape morphing in response to diverse environmental stimuli and programming intricate 3D shape morphing utilizing xerogel, LIG, and whole-material patterning. This technique has facilitated the development of untethered soft millirobots, with a minimum line width of 30 μm achieved during the patterning process. Notably, this approach has enabled the precise programming of various gestures, including kirigami, pop-ups, and bistable structures, thereby serving as adaptive switches for logic control circuits. To comprehend the underlying mechanism, we devised a theoretical model and conducted FEA to predict and design the intricate 3D shape morphing behavior. The strategic decoupling of shape morphing allows for the independent programming of each transformation step in response to different environmental stimuli. While this manufacture approach specifically decouples the response to magnetic field from the combined effects of humidity and temperature, it still enhances the DOF and functional capabilities of the MSSMs, enabling them to autonomously execute more complex tasks. As a proof-of-concept, we successfully implemented a scorpion-shaped MSSM that demonstrated continuous transformation during locomotion. Leveraging their high programmability, these MSSMs were subsequently investigated for applications as circuit maintenance robots, highlighting their unique contributions to the field of robotics. Their physical intelligence and adaptability render them highly programmable soft robots capable of navigating diverse environments and undertaking complex tasks. This versatility positions them as promising entities for applications in environmental sensing, self-adaptivity, soft actuation, and soft electronics, thereby paving the way for exciting advancements in the realm of robotic technology.

## Materials and Methods

### LIG and the Transference to PDMS Film.

A uniform thin layer of LIG was generated on top of a polyimide (PI) film using a CO_2_ laser, which was then patterned with an ultraviolet (UV) laser after transference to a PDMS film if necessary. Rather than directly generating a patterned LIG layer through programming the design profile, which has to face the challenge of low-resolution and varied conductivity limited by the equipment, the uniformly generated LIG film demonstrated advantages in quality and repeatability (sheet resistance: 33 Ω/square) and facilitated easier transference. To achieve this, a single-side PI tape (63 μm, Kapton®, Electron Microscopy Sciences) was used as the carbon source, and a 150-W CO_2_ laser cutter (wavelength, 10.6 μm; beam size, ~120 μm, Universal Laser Systems, PLS6-150D) was employed to convert dense PI to porous graphene. The optimized parameters in the engraving mode for this LIG-based conductive layer include laser power—4.1 W, laser speed—260 mm/s, laser point-per-inch—1,000, and pulse repetition frequency (PRF)—10.5 kHz. Once the LIG-based conductive layer was engraved, an uncured magnetic elastomer composite (PDMS mixed with NdFeB hard magnetic microparticles by planetary stirrer in a 1:1 weight ratio; PDMS, 15:1 base to curing agent ratios in weight; NdFeB particles, 5 μm in average diameter, Magnequench GmbH, MQP-15-7) was coated onto the engraved PI film and cured at 80 °C for 6 h, during which the thickness of the PDMS membrane was controlled to approximately 120 μm using a spacer and razor. The uncured PDMS was able to penetrate into the pores of LIG, forming a strong bond that allowed for the complete transfer of the LIG layer onto the surface of the PDMS after peeling off from the PI tape.

### Electro-Crosslinking of Xerogel by LIG-Based Electrodes.

The LIG-PDMS-based conductive bilayer film was utilized for the electro-crosslinking of xerogel. Prior to this, plasma treatment was performed on the LIG-PDMS film for 10 min to enhance surface activity. Sodium alginate solution was then uniformly added on top of the LIG side, followed by 10 min of vacuuming to allow the alginate to penetrate the LIG surface. For electro-crosslinking, the LIG layer served as the bottom electrode, while an ITO glass was used as the top electrode. A 4 V DC voltage was applied for 120 s to initiate the polymerization process of alginate. Following this, the uncured solution was carefully removed by washing the composite elastomer with distilled water. Finally, the composite elastomer with the deposited alginate was left at room temperature for 48 h to allow for the evaporation of moisture in the alginate network. This process yielded a crosslinked alginate network within the LIG-PDMS composite elastomer, which exhibited tunable and reversible swelling behavior in response to temperature and humidity changes.

### Laser Engraving-Based Robot Programming.

An UV laser-based high-resolution engraving method was introduced to decouple the multi-stimuli-responsive region, program the xerogel-introduced deformation and locomotion, and selectively tune the stiffness of joints for bending in soft robotics. This method demonstrated significant potential for the development of soft robotics with enhanced performance capabilities. The optimized engraving parameters allowed for the selective removal of specific layers, enabling precise control of the soft robot's motion and deformation. For selectively removing the xerogel layer, the optimized engraving parameters included a laser power of 0.25 W, a laser speed of 650 mm/s, and some other settings, with a PRF of 10.5 kHz. Furthermore, for selectively removing both the xerogel and the 60 µm PMDS layer, where the soft joints existed, the optimized engraving parameters were a laser power of 2.3 W, a laser speed of 600 mm/s, and some other settings, with a PRF of 10.5 kHz. Additionally, the proposed engraving method allowed for precise control over the amount of material to be removed by adjusting the power and speed of the laser system.

### Laser Cutting–Based Robot Programming.

In addition to the previously mentioned engraving method, laser cutting–based programming proved to be an efficient approach for programming soft robotics with desired functions. It allowed for xerogel-induced deformation by altering both the principal stress direction and the local body stiffness of the soft robot. After preparing the multiresponsive material sheet, a UV laser was employed to pattern the sheet into the desired shape using a cutting mode, achieving a minimum linewidth of 20 µm, commonly used in pop-up and bending deformation designs. The optimized engraving parameters for this cutting-based programming method included a laser power of 3.5 W, a laser speed of 280 mm/s, and a PRF of 10.5 kHz. Notably, to ensure the functionality and performance of the soft robot, both engraving and cutting-based programming methods were typically used together in different regions of the soft robot for various purposes. This ensured that the proposed soft robot could respond appropriately and efficiently to external stimuli. The operations, increasing the RH to 90% at room temperature and then increasing the temperature at RH 30%, were used to transform the MSSMs.

### Magnetic Profile Programming and Electromagnetic Control.

A jig-assisted method was used for encoding the desired magnetic profile M(xy) of the ferromagnetic soft robot. To obtain the desired magnetization profile, the soft robot was placed into the jig with a series of cutout parts in designed shapes. The soft robot was then magnetized by applying a large magnetization field B (1.8 T) in the +x direction inside a vibrating sample magnetometer (EZ7 VSM, MicroSense LLC). A uniform magnetic field with a maximum strength of 11 mT in 3D space was generated by a coil setup within a workspace with a size of 4 × 4 × 4 cm^3^. The magnetic field was controlled by modulating the supplied currents on the electromagnetic coils using six independent motor driver units (SyRen25) and an Arduino microcontroller operating at 1.2 kHz. The robot’s motion was tracked using two cameras (Basler, aCa2040-90uc). The first camera operated at 120 frames per second (fps) with a frame size of 2,040 × 1,020 pixel^2^ and was placed orthogonal to the *Y*-*Z* plane of the workspace to observe the robot from the side. The second camera operated at 60 fps with a frame size of 2,040 × 1,400 pixel^2^ and had a top view observing the *X*-*Y* plane of the workspace through a mirror placed at an angle of 45° above the test surface.

### FEA.

FEA was conducted using the commercial software ABAQUS to calculate the deformations of the multilayer soft robot. To model the ultrathin geometry of the soft robot, a composite bilayer (xerogel and magnetic PDMS layer) shell element was used, with refined meshes to ensure computational accuracy. As the induced strains during bending and twisting deformations were relatively small, the linear, elastic constitutive relations were employed for the two material components (xerogel and magnetic PDMS composite). The LIG layer, which served as the bonding connector, was excluded from this model due to its porous internal configuration and low modulus. The thermal expansion coefficient was used instead of the hygroscopic expansion coefficient to maintain consistency with the experimental conditions. The orthotropic coefficient of thermal expansion of each material was exploited to predict orthotropic deformations. During the simulations, the temperature changes, measured with a thermal camera (FLIR T865), were applied to the whole-shell model, which induced the strain mismatch and resulted in the bending and twisting deformations. The parameters of magnetic PDMS composite include a Young’s modulus of 0.8 MPa, Poisson’s ratio of 0.5, and a thickness of 120 μm. The parameters of xerogel include a Young’s modulus of 3,750 MPa, Poisson’s ratio of 0.5, an orthotropic thermal expansion ratio of 0.005 in the principal direction, and a thickness of 2 μm.

## Supplementary Material

Appendix 01 (PDF)

Movie S1. Stripe-shaped structure shape morphing into various direction based on partial xerogel removal.

Movie S2. Stripe-shaped structure shape morphing into various direction based on patterned LIG layer.

Movie S3. Reprocess the shape morphing from spiral structure to U-shaped structure.

Movie S4. Shape morphing of albuca-shaped structure.

Movie S5. Light induced shape morphing of hand gestures.

Movie S6. Stripe-shaped structure shape morphing into various direction based on whole material removal.

Movie S7. Complex 3D shape morphing of tetraloop, and Chichen Itza structures.

Movie S8. Shape morphing of pop-up structures.

Movie S9. Decoupled control of the multi-stepped bistable shape morphing.

Movie S10. Scorpion-shaped MSSM conducts shape morphing during the locomotion.

Movie S11. Logical control of the circuit with LED bulbs.

Movie S12. MSSM conducts the circuit repairing task.

## Data Availability

All study data are included in the article and/or supporting information.
