# Supplementary material for: Single-step precision programming of decoupled multiresponsive soft millirobots
Source: Proc Natl Acad Sci U S A. 2024 Mar 21;121(13):e2320386121. doi: 10.1073/pnas.2320386121 (PMC10990116; doi:10.1073/pnas.2320386121)
Supplement: Supplementary file 1 — Appendix 01 (PDF) [file pnas.2320386121.sapp.pdf]

PNAS

Supporting Information for
Single-Step Precision Programming of Decoupled Multi-Responsive Soft Millirobots

Zhiqiang Zheng[1#], Jie Han[1,4,5#], Qing Shi[2,3#], Sinan Ozgun Demir[1], Weitao Jiang[4,5], Metin Sitti[1,6,7*]

* Corresponding author: Metin Sitti
Email: sitti@is.mpg.de

This PDF file includes:

Note of Mechanical Analysis
Figures S1 to S13
Legends for Movies S1 to S12

Other supporting materials for this manuscript include the following:

Movies S1 to S12

Supporting Information Text

Mechanical Analysis. Based on Fig. 2A, the width ratio of the xerogel area and xerogel-removed microgroove area is represented as:

$$\Delta W = \frac{W_{\text{xerogel}}}{W_{\text{microgroove}}} \quad (1)$$

where the whole width is represented as:

$$W = W_{\text{xerogel}} + W_{\text{microgroove}} \quad (2)$$

$$W_{\text{microgroove}} = \alpha W \quad (3)$$

$$\alpha = \frac{1}{1+\Delta W} \quad (4)$$

Euler-Bernoulli beam theory was used in analysing the deformation of the sample beam, and we assume that the thickness and across section have no change during the shape morphing as shown in the following figure.

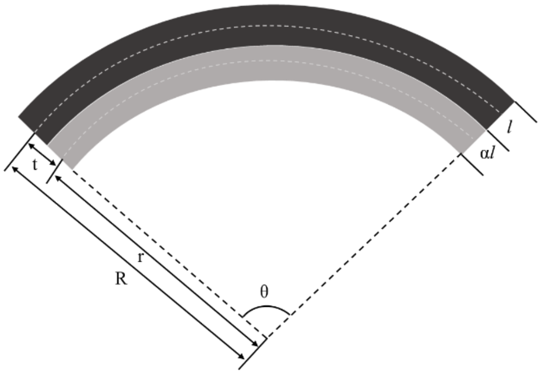

In the figure, l and αl are the length of the passive and active layers respectively. α is the shrinkage ratio. R and r are the radius of the passive and avtive layers. θ is the bending angle of the beam structure. t is the half thickness of the whole structure.

$$r\theta = \alpha l \quad R\theta = l \quad R - r = t \quad (5)$$

$$(1-\alpha)l = t\theta \quad (6)$$

Thus, the curvature (K) can be represented as:

$$K = \frac{2(1-\alpha)}{t(1+\alpha)} \quad (7)$$

The deformation mechanism of the pop-up and bistable structures is shown as the following figure.

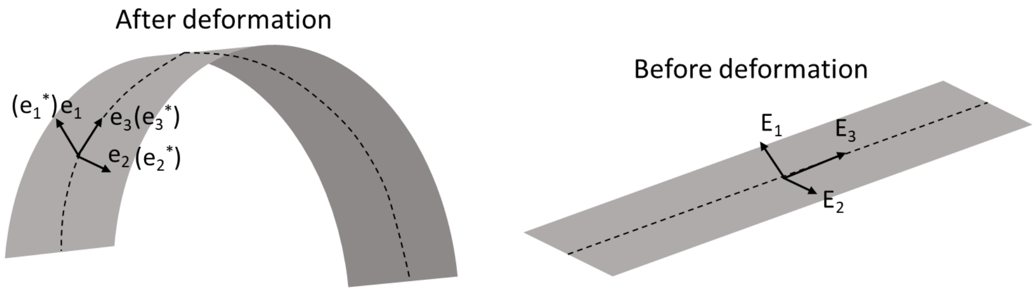

The Frenet frame of the central line is $\vec{e}_i^{\,*}$ and the Euler frame of the curved beam after deformation is $\vec{e}_i$. In the buckling deformation, the $\vec{e}_i^{\,*} = \vec{e}_i$ if there is no torsion at the across section. We assumed the angle between the $\vec{e}_i^{\,*}$ and $\vec{e}_i$ is β. Thus,

$$\begin{bmatrix} \vec{e}_1 \\ \vec{e}_2 \\ \vec{e}_3 \end{bmatrix} = \begin{bmatrix} \cos\beta & \sin\beta & 0 \\ -\sin\beta & \cos\beta & 0 \\ 0 & 0 & 1 \end{bmatrix} \begin{bmatrix} \vec{e}_1^{\,*} \\ \vec{e}_2^{\,*} \\ \vec{e}_3^{\,*} \end{bmatrix} \tag{8}$$

As the curvature of the beam structure is described as K_1 and K_2, the curvature K_1 and K_2 can be expressed based on the Euler Frame,

$$K_1 = \frac{d\vec{e}_3}{dS}\vec{e}_2 \qquad K_2 = \frac{d\vec{e}_3}{dS}\vec{e}_1 \tag{9}$$

As S is the arc length and $\vec{r}$ is the coordinate of the central axis, we have

$$\vec{e}_3^{\,*} = d\vec{r} / dS \tag{10}$$

$$d\vec{e}_3^{\,*} / dS = | d\vec{r} / dS | = K_1^* \vec{e}_1^{\,*} \tag{11}$$

Thus,

$$K_1 = -\frac{d\vec{e}_3^{\,*}}{dS}(-\sin\beta\vec{e}_1^{\,*} + \cos\beta\vec{e}_2^{\,*}) = \sin\beta K_1^* \tag{12}$$

$$K_2 = -\frac{d\vec{e}_3^{\,*}}{dS}(\cos\beta\vec{e}_1^{\,*} + \sin\beta\vec{e}_2^{\,*}) = \cos\beta K_2^* \tag{13}$$

Since β = 0, we have K_1 = 0 and $K_2 = K_1^*$, which means that the curvature along the direction ($\vec{e}_2$, $\vec{e}_3$) are always 0, and the curvature along the direction ($\vec{e}_1$, $\vec{e}_3$) always equal to the curvature of the central line.

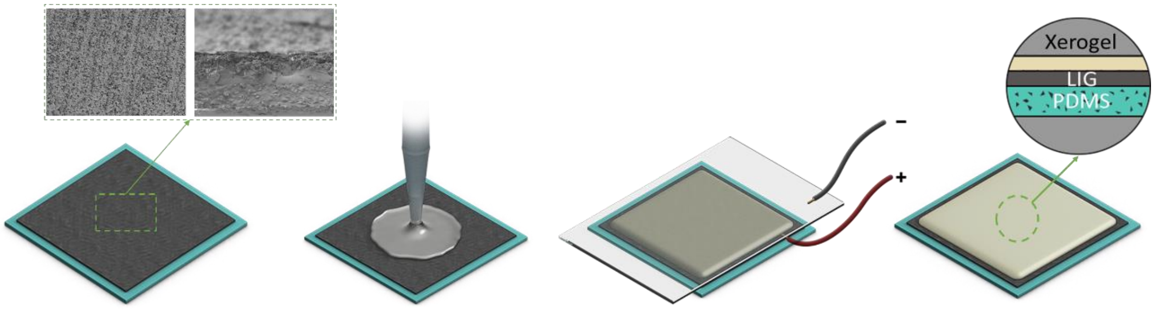

Fig. S1. The MSSM fabrication process using a multi-layered material. Laser was used to generate the conductive layer of LIG onto PI tape. Then, the uncured PDMS and NdFeB hard magnetic microparticles were mixed in a 1:1 weight ratio and poured on the top of the graphene surface to generate LIG coated PDMS. Electrodeposition solution was deposited between two electrodes onto the LIG, which was dried in a dark and damp place (xerogel layer) for further processing. The SEM images gives the top and side view of the LIG-PDMS structure.

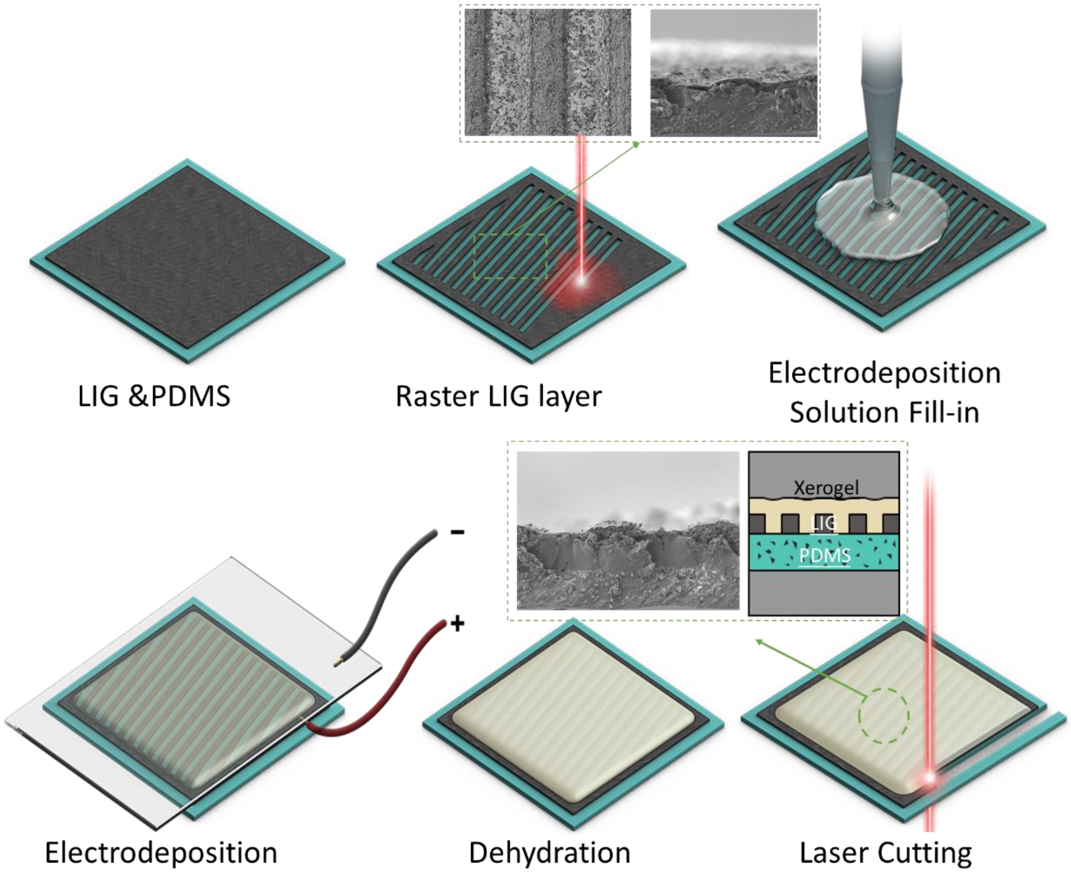

Fig. S2. The MSSM fabrication process with a patterned LIG layer. Before the electrodeposition, the laser was used to generate grid pattern on the LIG layer. After the electrodeposition and dehydration, the pattern MSSM can be formed. The SEM images gives the top view of laser patterned LIG surface and side view of the laser patterned LIG-PDMS and after xerogel coated structure.

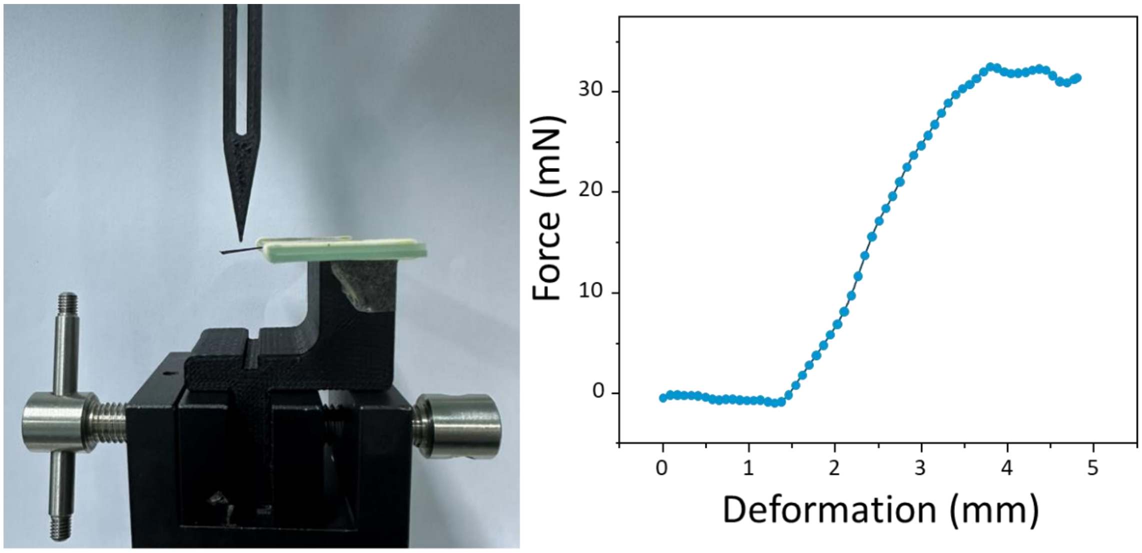

Fig. S3. The setup and test result of stiffness. We tested the press bending performance of the double side strip structure with 10 mm length and 4 mm width. In the environment of RH 30% and temperature 20°C, the force test result is shown as follow. After the calculation, the bending stiffness is 25.76 mN/mm^2.

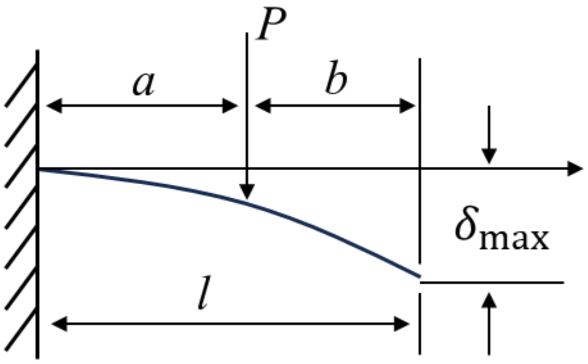

$$k = EI = \frac{Pa^2}{6\delta_{\max}}\left(3l - a\right)$$

Where k is the bending stiffness. E is the elastic modulus. I is the second moment of area about the neutral axis. l is the length of the beam. P is the stress. $\delta_{\max}$ is the maximum displacement of the beam. a is the distance from boundary to stress.

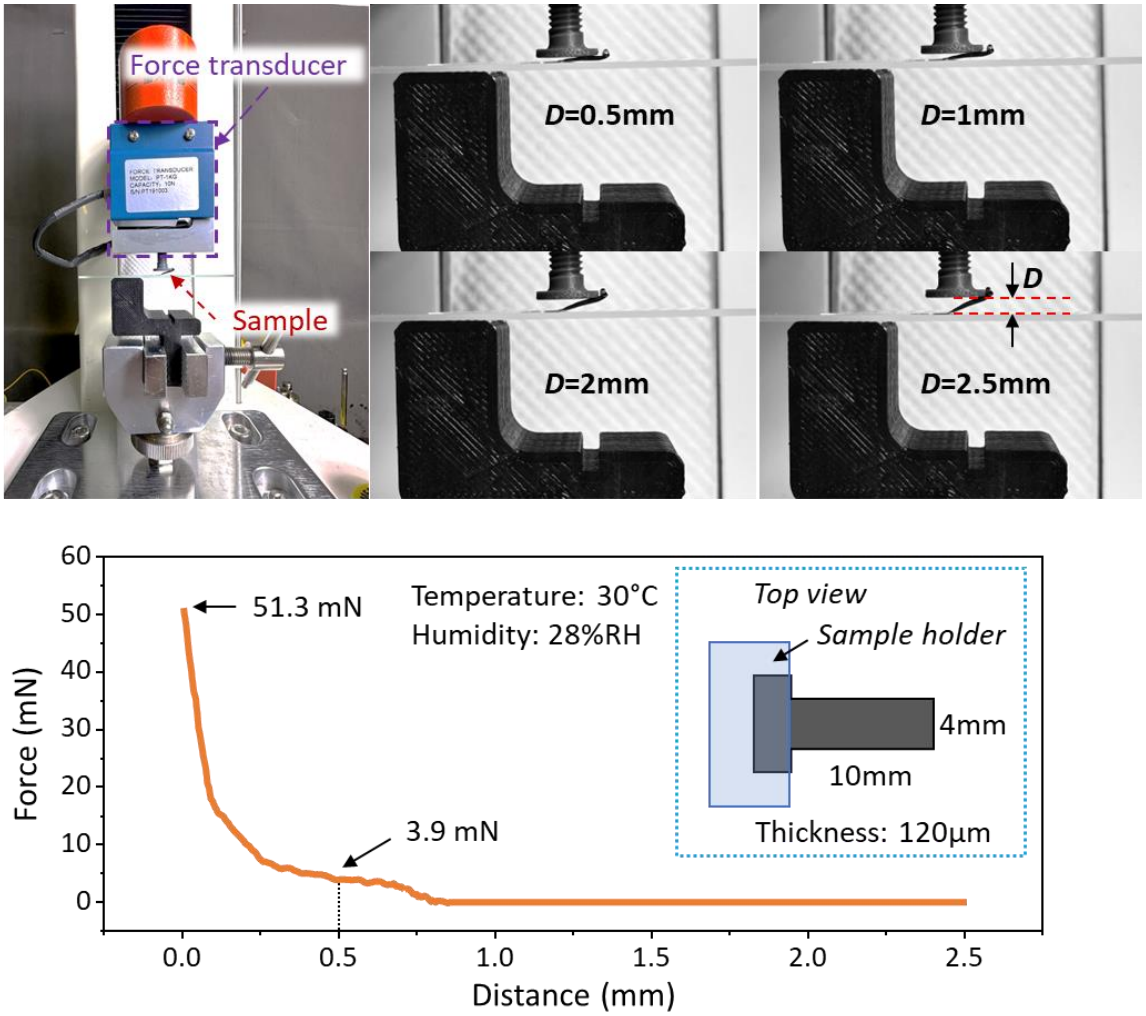

Fig. S4. The setup and test result of bending force. We tested the press bending performance of a strip structure with 10 mm length and 4 mm width. In the environment of RH 28% and temperature 30°C. The bending force is up to 51.3 mN. We used the force transducer to slowly move to the bended strip structure until contact to the stage under a quasi-static condition (0.5 mm/min). Based on this method, the last data point where distance set as 0 mm is the maximum bending force that can be generated by the strip structure.

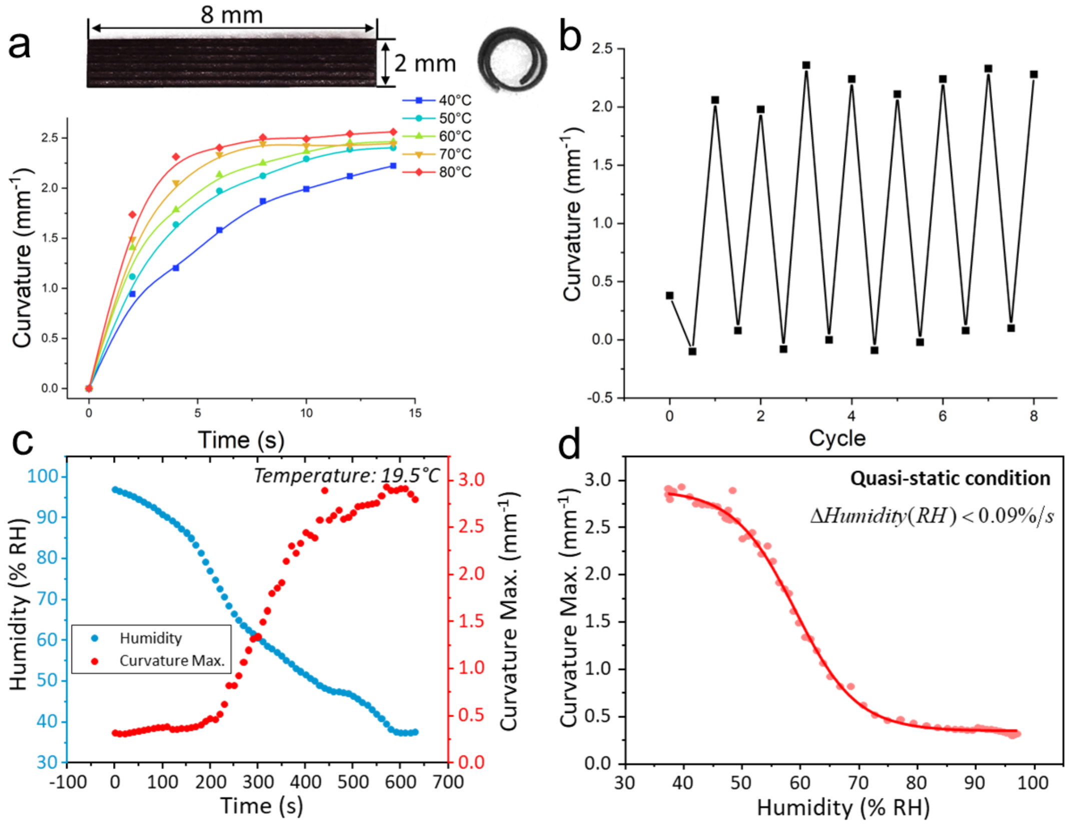

Fig. S5. The transformation speed and stability of the strip structure. (a) Time-dependent curvature as function of environmental temperature varying from 40°C to 80°C. (b) Transformation cycle of stripe structure in RH range 30% to 95% and temperature range 60°C to 20°C. (c)Time-dependent curvature and humidity change in the environmental temperature 19.5°C. (d) RH-dependent maximum curvature in the environmental temperature 19.5°C. In the fig. S6 (a), we transfered the strip structure from temperature 20°C and RH 90% to the hot plate with RH 30% temperature varying from 40°C to 80°C. And then, we tested the time of this transfermation process.

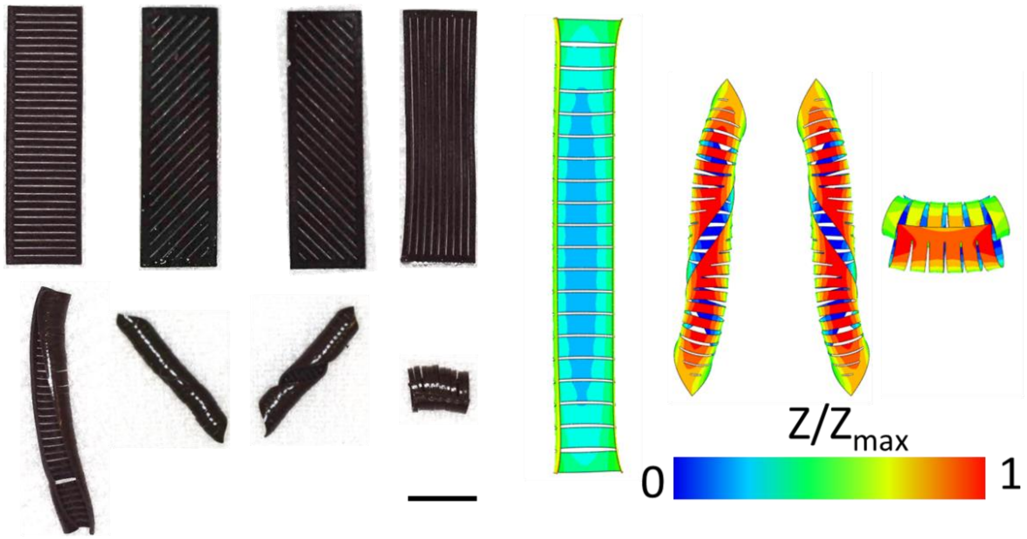

Fig. S6. The finite element analysis results of programming shape morphing with whole material patterning. Scale bar: 2 mm.

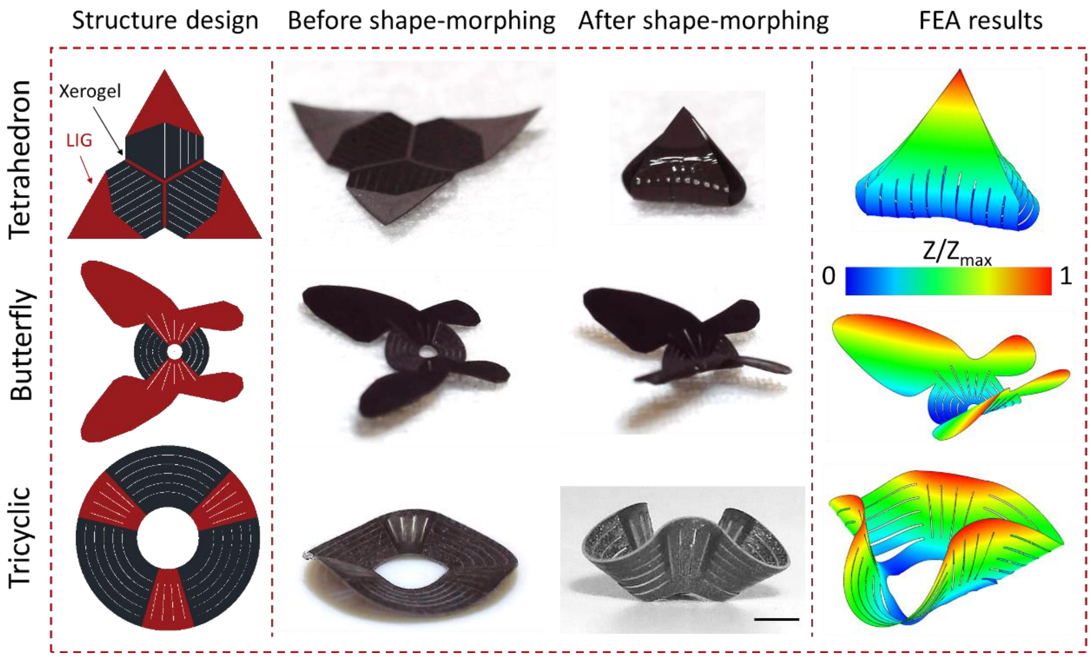

Fig. S7. The demonstrations and simulation results of various complex shape morphing kirigami structures. The programmed soft robots with complex shapes include tetrahedron, butterfly, and tricyclic. Scale bar: 1 mm.

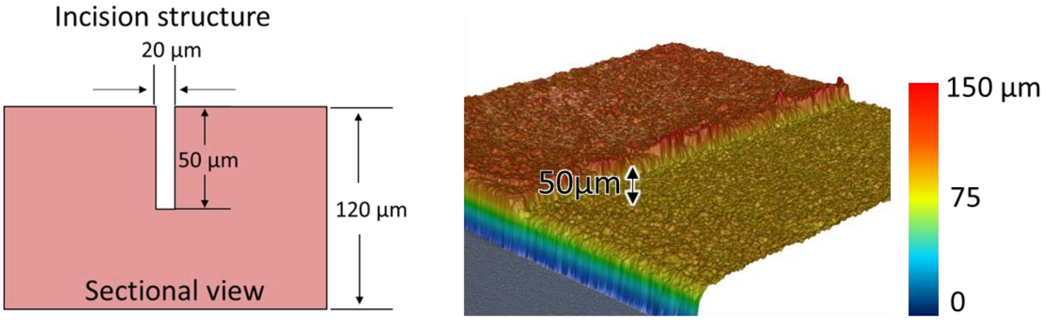

Fig. S8. The schematic (left) and 3D laser scanning image (right) of the incision structure generated by laser cutting.

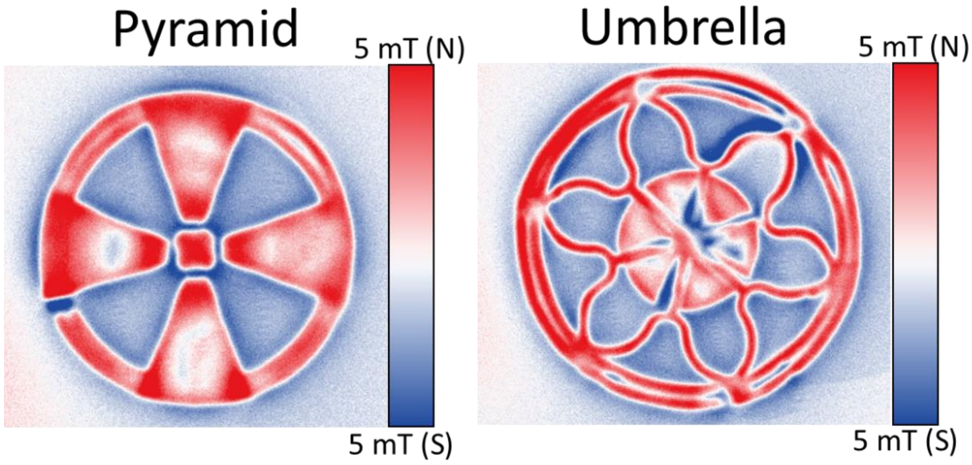

Fig. S9. The magnetic profile of pyramid and umbrella bistable structures measured by CMOS-MagView S (Matesy GmbH, Germany).

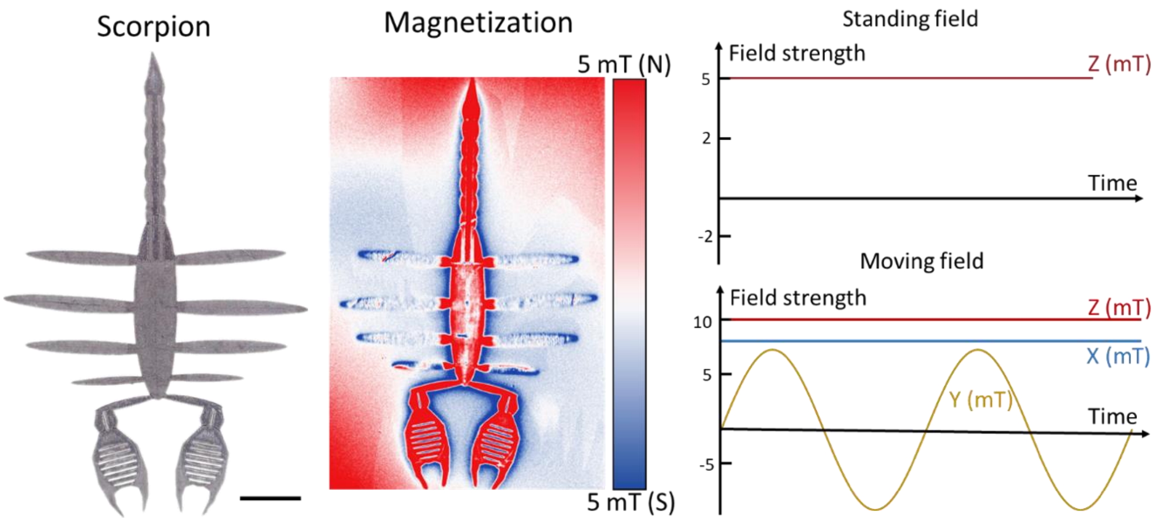

Fig. S10. The optical image (left), magnetic profile (middle) and actuation signal (right) of the scorpion-shaped MSSM. Scale bar: 1 mm.

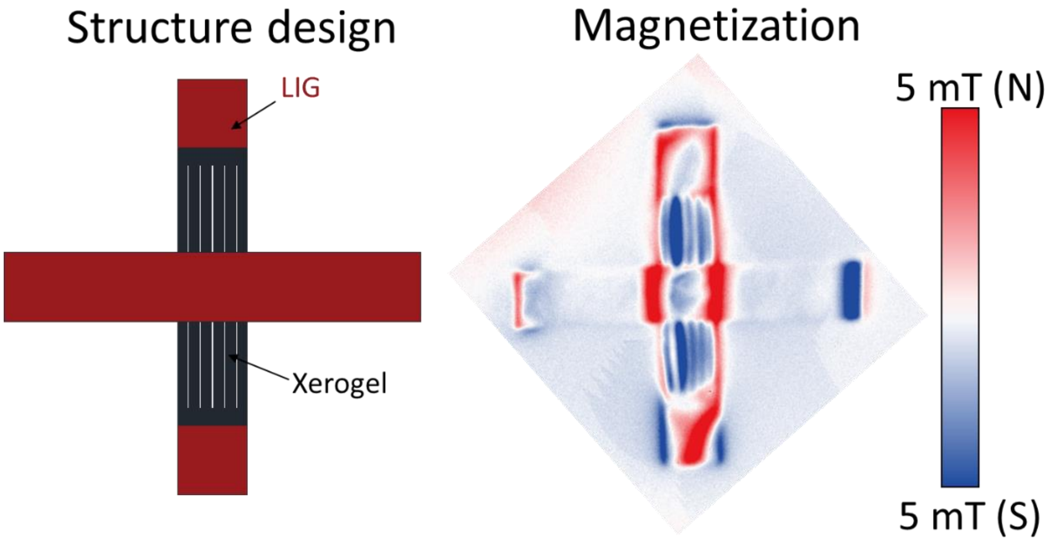

Fig. S11. The detailed structure design and magnetic profile of the environmentally adaptive switch.

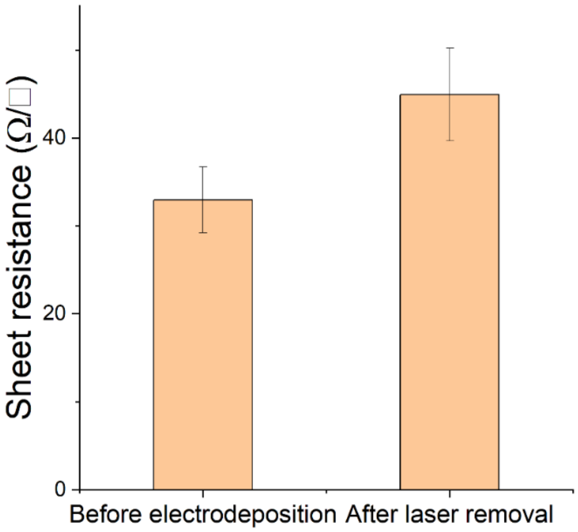

Fig. S12. The sheet resistance of the LIG layer before the electrodeposition and after the laser removal.

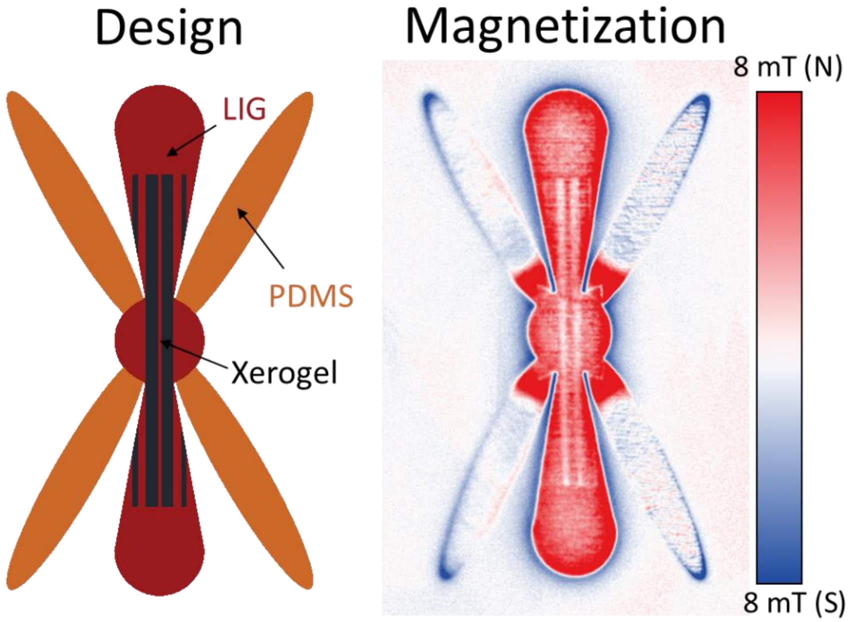

Fig. S13. The detailed structure design and magnetic profile of the circuit-maintaning MSSM.

Legends for Movie S1-S12

Movie S1. Stripe-shaped structure shape morphing into various direction based on partial xerogel removal.

Movie S2. Stripe-shaped structure shape morphing into various direction based on patterned LIG layer.

Movie S3. Reprocess the shape morphing from spiral structure to U-shaped structure.

Movie S4. Shape morphing of albuca-shaped structure.

Movie S5. Light induced shape morphing of hand gestures.

Movie S6. Stripe-shaped structure shape morphing into various direction based on whole material removal.

Movie S7. Complex 3D shape morphing of tetraloop, and Chichen Itza structures.

Movie S8. Shape morphing of pop-up structures.

Movie S9. Decoupled control of the multi-stepped bistable shape morphing.

Movie S10. Scorpion-shaped MSSM conducts shape morphing during the locomotion.

Movie S11. Logical control of the circuit with LED bulbs.

Movie S12. MSSM conducts the circuit repairing task.
